# What Drives Young Vietnamese to Use Mobile Health Innovations? Implications for Health Communication and Behavioral Interventions

**DOI:** 10.2196/mhealth.6490

**Published:** 2018-11-30

**Authors:** Bach Xuan Tran, Melvyn WB Zhang, Huong Thi Le, Hinh Duc Nguyen, Long Hoang Nguyen, Quyen Le Thi Nguyen, Tho Dinh Tran, Carl A Latkin, Roger CM Ho

**Affiliations:** 1 Institute for Preventive Medicine and Public Health Hanoi Medical University Hanoi Viet Nam; 2 Bloomberg School of Public Health Johns Hopkins University Baltimore, MD United States; 3 Biomedical Global Institute of Healthcare Research & Technology National University of Singapore Singapore Singapore; 4 Centre of Excellence in Behavioural Medicine Nguyen Tat Thanh University Ho Chi Minh City Viet Nam; 5 Institute for Global Health Innovations, Duy Tan University Da Nang Viet Nam; 6 Department of Hepatobiliary Surgery Vietnam-Germany Hospital Hanoi Viet Nam; 7 Department of Psychological Medicine Yong Loo Lin School of Medicine National University of Singapore Singapore Singapore

**Keywords:** youth, adolescent, Vietnam, mHealth, mobile phone, app

## Abstract

**Background:**

Mobile phone use in Vietnam has become increasingly popular in recent years, with youth (people aged 15-24 years) being one of the groups with the heaviest use. Health-related apps on mobile phones (mobile health [mHealth] apps) appear to be a feasible approach for disease and health management, especially for self-management. However, there has been a scarcity of research on mobile phone usage for health care among youth and adolescents.

**Objective:**

This study aims to identify the patterns of usage of mobile phone apps and the preferences for functionalities of mobile phone-based health-related apps among Vietnamese youth.

**Methods:**

An online cross-sectional study was conducted in Vietnam in August to October 2015. Web-based respondent-driven sampling technique was adopted to recruit participants. The online questionnaire was developed and distributed using Google Forms. Chi square and Mann-Whitney tests were used to investigate the difference in attitude and preference for mobile phone apps between the two genders.

**Results:**

Among 356 youths (age from 15 to 25 years) sampled, low prevalence was found of using mHealth apps such as beauty counseling (6.5%, 23/356), nutrition counseling (7.9%, 28/356), disease prevention (9.8%, 35/356), and disease treatment (7.6%, 27/356). The majority of users found the app(s) they used to be useful (72.7%, 48/356) and reported satisfaction with these apps (61.9%, 39/356). No significant differences were found between the genders in their perception of the usefulness of apps and their satisfaction with mobile health apps. Most of the participants (68.2%, 238/356) preferred apps which are conceptualized and designed to run on a mobile phone compared to Web-based apps, and 50% (176/356) preferred visual materials. Approximately 53.9% (188/356) reported that it was integral for the mobile phone apps to have a sharing/social network functionality. Participants with a higher perceived stress score and EuroQol-5 Dimensions (EQ-5D) index were significantly less likely to use mHealth apps.

**Conclusions:**

This study found a low proportion using mHealth-related mobile phone apps, but a high level of receptiveness and satisfaction among Vietnamese youth. Acceptance level and preferences toward mHealth apps as well as specifically preferred functionalities discovered in this study are essential not only in conceptualizing and developing appropriate mobile phone interventions targeting youth and adolescents, but also in the application of technically advanced solutions in disease prevention and health management.

## Introduction

The advancement of the mobile phone has greatly enhanced and expanded the computing capabilities and functionalities of mobile phones and mobile devices [1]. Individuals using mobile devices are no longer confined to calling or text-messaging functionalities, but now they can perform a myriad of computing tasks with mobile phone apps. In 2017, it was estimated that more than 2.3 billion people owned a mobile phone and this figure will increase to 2.9 billion at the end of 2020 [2]. Mobile health interventions have taken advantage of this expansion and have been increasingly employed in clinical and community settings to educate and support people to protect, monitor, and manage their health [3].

Young adults and adolescents are among the heaviest mobile phone users. In Sweden, 93% of adolescents have a mobile phone [4], while in the United States and China, this rate is approximately 73% and 82%, respectively [5,6]. Some previous literature emphasized the health problems related to mobile phone use and abuse among these groups, such as poor sleep quality [7], sedentary lifestyle [8], and increased risk of mental health issues [4,9]. However, the popularity of the mobile phone provides opportunities to engage youth and adolescents in innovative mobile health (mHealth) interventions that facilitate personal health care and self-management [10,11]. Sexual health, for example, can be taught effectively to adolescents via mobile phone apps [12]. Majeed-Ariss et al [13] suggested that mobile phone apps could be considered a new approach to support adolescents in their management of chronic conditions. Apps can be customized and individualized by applying behavioral theories and interactive platforms; therefore, youth and adolescents can find appropriate apps based on their specific health care needs [14].

Despite a growing body of literature about mHealth, research on young adults and adolescents has been constrained [13]. Most trials have targeted adult populations, which may not be applicable to young adults and adolescents, who possibly have different patterns of mobile phone usage [5]. Previous literature argued that low attrition and adherence rates among these populations might be due to problems with designing apps, but it was not clear how the youth and adolescents adopt new mobile phone apps [15]. Hermawati and Lawson [16] reviewed and found that only 22.5% of mHealth trials involved targeted populations in the development phase. Therefore, an understanding of the usage patterns and preferences for mobile phone apps among youth and adolescents is critical to developing mHealth interventions effectively [17].

In recent years, mobile phone use in Vietnam has become increasingly common thanks to their affordability [18,19]. In 2017, the number of mobile phone users was estimated at 28.9 million people and this was projected to reach 42.7 million in 2022 [20]. Among 90% of mobile phone users, mobile phones are the main devices that individuals use to access the internet. Prior research conducted in Vietnam has demonstrated that those aged between 15 and 35 years spend an average of 169 minutes daily on their mobile devices [21], mostly on accessing social networking sites [19]. Moreover, Vietnamese adolescents and youth now face various health problems, such as risk behaviors (eg, alcohol abuse, smoking, unprotected sexual activities), overweight/obesity, and mental health issues [22-26], which can be intervened by using health-related mobile phone apps. Nonetheless, there has been a scarcity of research on mobile phone usage for health care among youth and adolescents in Vietnam. Therefore, this study aimed to explore their patterns of usage of mobile phone apps, attitudes toward health-related apps, and preferences for the functionalities of apps.

## Methods

### Study Setting and Population

An online cross-sectional study was conducted in Vietnam from August to October 2015. People who met the following criteria were invited to enroll in the study: (1) age from 15 to 25 years, (2) currently living in Vietnam, (3) have an email or a social network account to invite their peers, (4) able to consent and participate in the study, and 5) have a mobile phone device. There were no other specific exclusion criteria.

### Web-Based Respondent-Driven Sampling Technique and Sample Size

The Web-based respondent-driven sampling (WebRDS) technique was utilized to recruit participants. WebRDS holds the potential to apply in public health studies. This method has demonstrated to be able to recruit a representative sample among youth in the United States [27]. Moreover, in a study among college students, it was found to boost the recruitment process up to 20 times compared to traditional RDS [28].

In this study, we selected the first waves (ie, core seeds) as representative of the diversity of the sampled population by taking into consideration their age, gender, and level of education. This strategy potentially initiates long recruitment chains of multiple waves of recruits that can ensure sample equilibrium being reached [29]. Firstly, 30 seeds from Hanoi Medical University, Vietnam National University, Hung Yen High School, and Phan Boi Chau High School were invited to participate in the study. The first two universities were used to recruit young adults, whereas the others were used for enrolling adolescents. We selected a respondent as a seed of the WebRDS if he/she also was a high-energy sociometric star and committed to being generative in recruiting their peers in the study [29]. The response rate was 100%. Participants in the seed group were informed that they needed to assist in the recruitment of other participants through their individual social network. They could not only invite people from their schools, but also from other schools. There was no predetermined duration with regard to the duration of recruitment. The recruitment was terminated only when the recruitment network was deemed to be no longer able to expand in size. A total of seven cases that were duplicated and three cases that did not meet the inclusion criteria (ie, did not answer at least 23 questions) were excluded. The total size of the sample for our study was 356 youths.

### Study Procedure

After being invited to enroll in the study, the seeds were sent a Web link that contained an eligibility screening on the first page, information about the study and the electronic informed consent on the second page, and the Web-based questionnaire on the last page. The average time to complete this questionnaire was 20 minutes. Those participants who had difficulties accessing the original Web link were provided with an alternative Web link to access the survey.

After finishing the survey, each seed was asked to recruit at least five other members to participate in the survey, and the incentives were topped up by the number of peers they referred to the survey (US $0.50 per peer). We allowed copying of the Web link to text messages and social network sites to refer peers. Double entries were identified and removed via the email address that they have entered or IP address from the internet network that they logged in from.

### Web Survey Design

The online questionnaire was developed and distributed using Google Forms, which met the security requirements as set forth by the ethical approval board for this study. The Web-based survey consisted of a total of 40 questions; the minimum number of questions participants needed to answer to be included was 23. We developed the questionnaire based on previous studies on preferences for functionalities of mHealth apps [30-33]. The researchers also included a logic check of the survey questionnaire to ensure that the data captured corresponded to the theme of the questions and was accurately captured in the backend database. To determine the feasibility and the reliability of the platform, the Web-based questionnaire survey was piloted among a group of 20 youths of varying ages and genders. The pilot group provided the investigators with recommendations to further optimize the online survey platform. Because only several minor changes were raised that did not affect the answers of people participating in the pilot study, we decided to include those youths in the final sample.

The Web-based questionnaire consisted of the following parts:

1. Sociodemographics and health status: including age, gender, educational status, marital status, and current living location. We also asked respondents to report their height and weight to compute body mass index (BMI). Their health-related quality of life (HRQOL) and perceived stress were measured using EuroQol-5 dimensions-5 levels (EQ-5D-5L) with EQ-5D index (ranging from –0.452 to 1; higher index indicates higher HRQOL) and the 4-item Perceived Stress Scale (PSS) with perceived stress score (ranging from 0 to 16 points; higher score indicates a higher level of stress).
2. Mobile phone usage pattern: we asked questions about whether they had a mobile phone with access to a wireless network (with or without 3G), operating system of their existing mobile phone devices, duration of ownership of a mobile phone device, time to use mobile phone per day (for calling, texting, and other purposes), self-reported level of proficiency in the usage of a mobile phone, and types of mobile phone apps they have ever downloaded and used.
3. Attitudes toward using health-related apps: information of interest included types of health-related apps they currently used (beauty counseling, nutrition counseling, disease prevention/treatment counseling, or others) and levels of usefulness and satisfaction regarding these apps. Beauty counseling apps refer to apps that provide advice, for instance, for using functional foods or dermatological interventions (not related to specific cosmetics).
4. Preferences for a mobile phone app: to determine preferences for functionalities of mobile phone health-related apps, the following questions were asked: how the mobile phone app was conceptualized (ie, with an addition Web-based version or mobile phone-based only), whether the app was comprised predominantly of text or images, whether the app allowed for any forms of sharing/social network, and whether the app had advertisements within it.

### Statistical Analysis

STATA software version 12.0 was used to analyze the data. Chi square, Fisher exact, and Mann-Whitney tests were used to explore the differences between means and proportions of characteristics of interest by gender. Multivariate logistic regression was used combined with a stepwise backward strategy to build a reduced model. A *P* value of less than .05 was set as the level of statistical significance.

## Results

A total of 356 individuals completed the Web-based questionnaires, of which 32.0% (n=114) were males. Most of the participants had a university education (83.1%, 289/356) and were single (80.6%, 286/356). Approximately 45.2% (160/356) lived as tenants (rent a house/room) whereas 28.5% (101/356) lived with their families. The mean BMI was 19.7 (SD 2.0) kg/m^2^; the mean EQ-5D index and stress score were 0.76 (SD 0.16) and 6.5 (SD 2.1), respectively (Table 1).

Table 2 highlights that the Android operating system was dominant (62.9%, 224/356). Approximately 49.4% (176/356) of participants had used mobile phones for less than 24 months and 58.1% (202/356) rated themselves as being intermediate proficiency. Music players and social networks were the most frequently downloaded and used apps with 61.5% (219/356) and 51.1% (182/356), respectively. The participants used their mobile phone devices mostly for texting and other activities such as gaming or watching movies with means of 2.1 (SD 10.5) and 3.1 (SD 9.3) hours daily, respectively.

**Table 1:** Sociodemographics and health status of respondents (N=356).

Sociodemographics and health status		Participants
Male, n (%)		114 (32.0)
**Age groups, n (%)**		
	<18	6 (1.7)
	18-22	196 (55.4)
	>22	152 (42.9)
**Education attainment, n (%)**		
	≤High school	12 (3.5)
	Vocation training	7 (2.0)
	College	30 (8.6)
	University	289 (83.1)
	Postgraduate	10 (2.9)
**Marital status, n (%)**		
	Single	286 (80.6)
	Living with spouse/partner	69 (19.4)
**Current living location, n (%)**		
	Rent	160 (45.2)
	Dormitory	55 (15.5)
	Living with family	101 (28.5)
	Living with relatives	35 (9.9)
	Others	3 (0.9)
Body mass index, mean (SD)		19.7 (2.0)
EuroQol-5 dimensions (EQ-5D) index, mean (SD)		0.76 (0.16)
Perceived stress score, mean (SD)		6.5 (2.1)

**Table 2:** Mobile phone use patterns among respondents.

Mobile phone use patterns		Female (n=242)	Male (n=114)	Total (N=356)	*P* value
**Operating system, n (%)**					
	Android	158 (65.3)	66 (57.9)	224 (62.9)	.18
	iOS	45 (18.6)	33 (29.0)	78 (21.9)	.03
	Window Phone	32 (13.2)	18 (15.8)	50 (14.0)	.52
	Blackberry	4 (1.7)	2 (1.8)	6 (1.7)	.95
**Duration of using a mobile phone (months), n (%)**					
	<12 months	60 (24.8)	29 (25.4)	89 (25.0)	.19
	12 to <24 months	61 (25.2)	26 (22.8)	87 (24.4)	
	24 to <36 months	59 (24.4)	19 (16.7)	78 (21.9)	
	≥36 months	62 (25.6)	40 (35.1)	102 (28.7)	
**Level of proficiency in using the mobile phone, n (%)**					
	Novice	22 (9.3)	7 (6.3)	29 (8.3)	.008
	Intermediate	146 (61.6)	56 (50.5)	202 (58.1)	
	Advance	69 (29.1)	45 (40.5)	114 (32.8)	
	Expert	0 (0.0)	3 (2.7)	3 (0.9)	
**Apps downloaded and used (except mHealth apps), n (%)**					
	Games	98 (40.5)	67 (58.8)	165 (46.4)	.001
	Weather forecast	47 (19.4)	25 (21.9)	72 (20.2)	.58
	Music player	149 (61.6)	70 (61.4)	219 (61.5)	.98
	Movie player	58 (24.0)	37 (32.5)	95 (26.7)	.09
	Geographic Information system	49 (20.3)	34 (29.8)	83 (23.3)	.04
	Social networks	116 (47.9)	66 (57.9)	182 (51.1)	.08
	Financial management	11 (4.6)	11 (9.7)	22 (6.2)	.06
	News	78 (32.2)	49 (43.0)	127 (35.7)	.05
	Entertainment	81 (33.5)	43 (37.7)	124 (34.8)	.43
	Education	71 (29.3)	40 (35.1)	111 (31.2)	.28
	Sport	13 (5.4)	41 (36.0)	54 (15.2)	<.001
	Book	105 (43.4)	44 (38.6)	149 (41.9)	.39
	Shopping	29 (12.0)	6 (5.3)	35 (9.8)	.05
**Time to use mobile phone per day (hours), mean (SD)**					
	For calling	0.7 (0.7)	0.9 (1.5)	0.8 (1.0)	.34
	For texting	2.2 (11.0)	2.1 (9.4)	2.1 (10.5)	.19
	For others (eg, game, movies)	2.1 (2.5)	5.2 (16.0)	3.1 (9.3)	.07

**Table 3:** Usage, attitudes, and preferences for mobile phone health care apps.

Usage, attitudes, and preferences for mobile phone health care apps		Female (n=242), n (%)	Male (n=114), n (%)	Total (N=356), n (%)	*P* value
Using any health care apps		47 (19.4)	20 (17.5)	67 (18.8)	.67
**Types of health care apps**					
	Beauty counseling	20 (8.3)	3 (2.6)	23 (6.5)	.04
	Nutrition counseling	19 (7.9)	9 (7.9)	28 (7.9)	.99
	Disease prevention counseling	25 (10.3)	10 (8.8)	35 (9.8)	.65
	Disease treatment counseling	17 (7.0)	10 (8.8)	27 (7.6)	.56
**Useful for health**					
	Not useful	13 (28.3)	5 (25.0)	18 (27.3)	.79
	Useful	33 (71.7)	15 (75.0)	48 (72.7)	
**Satisfaction with mobile health apps**					
	Dissatisfied	19 (43.2)	5 (26.3)	24 (38.1)	.21
	Satisfied	25 (56.8)	14 (73.7)	39 (61.9)	
	Preferences				
**Type of apps**					
	Web-based apps	69 (29.2)	42 (37.2)	111 (31.8)	.14
	Mobile phone apps	167 (70.8)	71 (62.8)	238 (68.2)	
**Information and contents of apps**					
	Visuals	124 (51.9)	52 (46.0)	176 (50.0)	.30
	Text	6 (2.5)	1 (0.9)	7 (2.0)	
	Combination of visuals and text	109 (45.6)	60 (53.1)	169 (48.0)	
**Areas of apps**					
	Specific area (focus on one topic)	115 (48.7)	67 (58.8)	182 (52.0)	.08
	Integrative areas (focus on multiple topics)	121 (51.3)	47 (41.2)	168 (48.0)	
**Sharing/social network functionalities**					
	Yes	131 (55.0)	57 (51.3)	188 (53.9)	.52
	No	107 (45.0)	54 (48.7)	161 (46.1)	
**Feeling toward advertisements within apps**					
	Neutral	49 (20.5)	24 (21.6)	73 (20.9)	.70
	Negative	188 (78.7)	85 (76.6)	273 (78.0)	
	Positive	2 (0.8)	2 (1.8)	4 (1.1)	

Table 3 illustrates that 18.8% (67/356) had ever used any health care apps. Rates of using mHealth apps such as beauty counseling (6.5%, 23/356), nutrition counseling (7.9%, 28/356), disease prevention (9.8%, 35/356), and disease treatment (7.6%, 27/356) were also observed. The majority of users found the app(s) they used to be useful (72.7%, 48/356) and reported satisfaction with such app(s) (61.9%, 39/356). There were no significant differences between the genders in their perception of the usefulness of apps and their satisfaction with mobile health apps. There was a significant difference between genders in their usage of beauty counseling apps, with there being a predominance of females over males using beauty counseling apps (*P*=.04). Most participants (68.2%, 238/356) preferred apps which were conceptualized and designed to run on a mobile phone as compared to Web-based apps, and 50.0% (176/356) preferred apps with a dominance of visuals. Approximately 53.9% (188/356) reported that it was integral for the mobile phone apps to have a sharing/social network functionality, and 78.0% (273/356) felt negatively about the advertising within apps.

Table 4 depicts that those studying in college were more likely to use health-related apps (OR 4.16, 95% CI 1.76-9.82) compared to those having a high school education or less. Meanwhile, people having a higher stress score or EQ-5D index and an intermediate level of using the mobile phone were less likely to download this type of app.

**Table 4:** Associated factors with the use of mobile phone health-related apps.

Using any health-related apps		Odds ratio (95% CI)	*P* value
**Current living location (vs homestay)**			
	Living with relatives	0.30 (0.08-1.12)	.07
**Education (vs ≤high school)**			
	College	4.16 (1.76-9.82)	<.001
**Duration of mobile phone use (vs <12 months)**			
	12 to <24 months	1.61 (0.81-3.19)	.18
**Level of proficiency in using the mobile phone (vs novice)**			
	Intermediate	0.26 (0.10-0.69)	.01
	Advanced	0.48 (0.18-1.29)	.15
Perceived stress score		0.86 (0.74-0.99)	.04
EuroQol-5 dimensions (EQ-5D) index		0.13 (0.02-0.84)	.03

## Discussion

This study critically points out the mobile phone app usage patterns and preferences that could direct further mHealth interventions among Vietnamese youth. Although we only observed a low proportion of respondents using mHealth apps, we found a positive premise to develop such apps since the majority of youths using mHealth apps expressed their high degree of acceptance and satisfaction. This study also implies some important features regarding app conceptualization and design, which could help to optimize the receptiveness of mHealth apps in Vietnamese youth.

Among a sample of Vietnamese youths, we found a low percentage of people using mHealth-related mobile phone apps (18.8%, 67/356), from 6.5% (23/356) for beauty counseling apps to 9.8% (35/356) for disease prevention counseling apps. These figures were similar to results reported in previous reports in Vietnam and other countries, in which health-related apps only accounted for a minor percentage of apps downloaded [13,15,21]. To our knowledge, there are several reasons that can be used to explain this phenomenon. First, the youth might be more attracted by the apps that can entertain them after learning or working time rather than health-related apps. Second, there is a lack of mHealth apps in the Vietnamese language, which would cause difficulties for youth and adolescents in navigating the content (in a foreign language) and deciding which apps are the most suitable for them. Meanwhile, the few existing health-related apps developed by Vietnamese firms available in app stores such as eDoctor and Bacsi24 mainly have been used to connect health care facilities and patients, and lack specific content for youth and adolescents. Finally, most existing mHealth apps have not had their effectiveness evaluated nor have been officially approved by health care authorities, which could undermine the acceptability of the public. Currently, the Vietnam Ministry of Health has drafted a policy to regulate the implementation of distance health care (including the dissemination of mHealth apps). Quality assurance from a government body would be expected to increase the demand for health-related apps among Vietnamese youth and adolescents.

Despite the low download rates of health-related apps, the majority of people who actually downloaded and used these apps reported finding the apps useful (72.7%, 48/356) and were satisfied with their decision (61.9%, 39/356). This result is more positive than what was indicated in an American study, which reported that most participants who downloaded mHealth apps condemned them as irrelevant and/or user unfriendly and did not utilize the downloaded apps [15]. Such high satisfaction found in our study may be the result of a number of factors—the relevance of existing health apps, open-mindedness and acceptability of these particular youth and adolescents regarding mHealth apps, or possibly a high level of concern toward health among these particular individuals—but it suggests that mHealth apps can be accepted and helpful for users, perhaps with content enhanced to be more specific and design improved to be more youth appealing.

Enhancing the content of the mHealth apps would be of great importance in changing the behavior of people toward using these apps. Our study found an inverse relationship between perceived stress score and the EQ-5D index of participants against the likelihood of mHealth app utilization, implying a possible lack of relevant and perceived useful content and function within apps for both perceived healthy and stress-suffering individuals. Existing studies on mHealth have highlighted a major issue of mHealth apps not having been built based on evidence from sufficiently large empirical studies nor having health professionals involved in the development process [13]. Utilizing the knowledge and expertise of health professionals in building the mHealth apps would not only ensure the quality of app content, but also likely increase the acceptability of users knowing that the apps have been backed by more reliable professional know-how.

Incorporating other content into mHealth platforms such as games, music players, and social networks, which we found in our study to be the three most downloaded types of apps, would also likely help increase app acceptability and usage. Primack et al [34] found that electronic games could provide educational health messages and motivate adolescents and young adults to do physical activity and self-manage their health conditions. Meanwhile, social network/sharing functionalities would enable users to share information/achievements onto their own personal social networks and facilitate learning from healthy lifestyle / keeping fit experiences, shared by their peers through similar platforms. Previous literature has shown that social networks and social influences have large impacts on behavior changes of Vietnamese youth [35] and are significant facilitators of mHealth app usage among adolescents and youths [15]. Indeed, the majority of our participants preferred having sharing/social network functions in their mHealth apps. Additionally, with regard to interface design, preference was found for visuals, which suggests the integration of multimedia with videos and images to elaborate apps would appeal to youth and adolescents [15].

Several implications can be drawn from this study. First, for researchers and developers, the involvement of end users and health professionals in developing and evaluating mHealth apps is essential. End users could inform their preferences regarding content, format, and display, as well as express their experiences in using and perception toward the apps, which are crucial for ensuring the relevance and appeal of the apps developed [36,37]. Meanwhile, health care professionals could facilitate the development and approval of these health-related apps, in particular those targeting disease prevention apps—the most downloaded types of mHealth apps as indicated by our results. Furthermore, the promising acceptability and usage of mHealth apps, coupled with the current popularity of mobile phone devices, social networks, and connection through the internet, suggests that physicians and health management authorities (eg, the Ministry of Health) should continue to pay more attention to this technology-enabled solution and consider it a key solution in health management and disease prevention. Support in terms of legal platforms and policies would encourage the creation and enhancement of apps by developers, while campaigns on benefits of the correct use of these health apps would help promote usage among the population, assisting self-management, early detection, and prevention of diseases.

The strength of this research is that we provided evidence about the usage patterns of mobile phone apps and identified user attitudes and preferences to conceptualize further mHealth apps. This type of study has not been conducted previously in Vietnam. However, there are several methodological limitations that should be acknowledged. First, due to the recruitment-via-internet method, our study might not be able to recruit people who do not have access to the internet, thus our sample cohort might not be entirely representative of the general population, which could undermine the ability to generalize our results. Moreover, our sample seems to be homogeneous, which makes it impossible to stratify our analysis into other demographic characteristics such as age and education. The WebRDS method produced shorter recruitment chains than expected, hence we could not establish equilibrium with this sample. Moreover, we did not develop a Web-based questionnaire that fits on tablets or mobile phones, which possibly impacted the ability to recruit more participants since these devices are increasingly popular. Furthermore, some features, namely input method, earning rewards, or connection between app users and health care providers, were not included in this study, suggesting further research is needed to provide comprehensive evidence about the preference for mHealth apps.

Finally, because we only researched general features of apps rather than particular content, such as physical activity [30], HIV [31], diabetes [32], or cystic fibrosis [33], some tailored features for these topics could not be studied. For example, patients with HIV required apps with automated reminders, motivational messages, mental support, and password protection [31]. Meanwhile, for apps promoting physical activity, Rabin and Bock [30] revealed that automated progress tracking, problem-solving messages, and user-friendly interfaces were those features most preferred. Most of the studies also highlighted some features that should be included for mHealth apps, such as communicating with health care providers and location tracking for contextualized messages [31-33]. Therefore, it appears that specific features for each condition and each population should be considered independently during development of mHealth apps.

This study found low proportions using mHealth-related mobile phone apps, but a high level of receptiveness and satisfaction among Vietnamese youth. Acceptance level and preferences toward mHealth apps as well as specifically preferred functionalities discovered in this study are essential not only in conceptualizing and developing appropriate mobile phone interventions targeting youth and adolescents, but also in the application of technically advanced solutions in disease prevention and health management.

## Abbreviations

BMI: body mass index
EQ-5D: EuroQol-5 dimensions
HRQOL: health-related quality of life
mHealth: mobile health
PSS: Perceived Stress Scale
RDS: respondent-driven sampling
WebRDS: Web-based respondent-driven sampling
